# Tumor-responsive cuproptosis nanoinducer realizing efficient PANoptosis for enhanced cancer immunotherapy

**DOI:** 10.7150/thno.115275

**Published:** 2025-08-16

**Authors:** Kaiqing Yun, Xiaohong Yu, Shuang Liang, Qingling Wang, Ziyi Zhang, Yue Han, Yueyang Zhao, Yuxuan Peng, Lang Rao, Yong Cui, Zhaohui Wang

**Affiliations:** 1 State Key Laboratory of Bioactive Substance and Function of Natural Medicines, Institute of Materia Medica, Chinese Academy of Medical Sciences & Peking Union Medical College, Beijing 100050, China.; 2 College of Medical Devices, Shenyang Pharmaceutical University, Shenyang 110016, China; 3 Beijing Key Laboratory of Drug Delivery Technology and Novel Formulation, Institute of Materia Medica, Chinese Academy of Medical Sciences & Peking Union Medical College, Beijing 100050, China.; 4 Institute of Chemical Biology, Shenzhen Bay Laboratory, Shenzhen 518132, China.; 5 School of Life Sciences and Biopharmaceutical Science, Shenyang Pharmaceutical University, Shenyang 110016, China.; 6 Wuya College of Innovation, Shenyang Pharmaceutical University, Shenyang 110016, China.; 7 Hebei Key Laboratory of Innovative Drug Research and Evaluation, School of Pharmaceutical Sciences, Hebei Medical University, Shijiazhuang 050017, China.

**Keywords:** Nano-inducer, PANoptosis, Cuproptosis, Regulated cell deaths, Cancer immunotherapy, Tumor-responsive nanoparticles

## Abstract

**Rationale:** The induction of multiple forms of regulated cell death (RCD) presents a promising approach for antitumor immunotherapy. However, the heterogeneous tumor microenvironment (TME) limits the efficacy of single-mode RCD induction strategies.

**Methods:** We engineered Elesclomol-Cu@PDPA nanoparticles (PEC NPs) designed to induce cuproptosis and subsequent PANoptosis. These NPs remain stable during circulation but rapidly dissociate in the acidic TME, releasing Cu^2+^ and Elesclomol to trigger cuproptosis.

**Results:** The cuproptosis induced by PEC NPs resulted in PANoptosis of tumor cells. This process stimulated immunogenic cell death and activated a systemic immune response by promoting immune cell infiltration and reprogramming the immunosuppressive TME. PEC NPs demonstrated potent tumor growth inhibition and exhibited a synergistic antitumor effect when combined with immune checkpoint blockade therapy.

**Conclusions:** This study provides a robust strategy utilizing PEC NPs to induce efficient cuproptosis and PANoptosis for enhanced immunotherapy. It offers new insights into boosting tumor immunogenicity through the activation of multiple RCDs pathways.

## Introduction

Immunotherapy has emerged as a promising approach in cancer treatment with unprecedented clinical success. However, their therapeutic effectiveness is limited in a broad range of solid tumors due to the immunosuppressive tumor microenvironment (TME) and insufficient activation of immune system, with only a subset of patients responding positively to treatment 1. Accumulating evidences have shown that cancer immunotherapy is crucially dependent on the efficient induction of tumor immunogenicity, particularly through the promotion of damage-associated molecular patterns (DAMPs) that released during cell death 2,3. This immunogenic cell death (ICD), defined as a type of regulated cell death (RCD) that plays a critical role in stimulating immune responses 4,5. Various forms of proinflammatory RCD, such as pyroptosis and ferroptosis, exhibiting ICD-like characteristics and act as adjuvants to boost antitumor immunity 6. Nonetheless, the inherent heterogeneity of tumors presents a serious challenge, as different cancer cell types exhibit varying sensitivities to specific form of cell death 7. Meanwhile, the frequent lacking or suppressed expression of critical genes involved in RCD pathways complicate the tumor killing effect 8,9. Therefore, simultaneously triggering multiple redundant pathways of RCD is able to comprehensively enhance the immunogenicity and sensitization of cancer cells through the release of DAMPs and inflammatory cytokines, which remains in the early stages of exploration.

The emerging concept of PANoptosis offers promising insights into the development of effective strategies to enhance immune responses 10. PANoptosis is an innate immune, lytic and inflammatory form of RCD that involves the activation of caspases and receptor-interacting protein kinases 11,12. This process integrates key features of pyroptosis, apoptosis, and necroptosis but cannot be fully characterized by any single modality of cell death 13. By disrupting cellular barriers and extensively releasing DAMPs, increasing evidence suggests that PANoptosis elicit a robust and long-lasting tumor-specific immune response 14,15. Current strategies to induce PANoptosis are limited to specific pathogens, DAMPs, pathogen-associated molecular patterns (PAMPs), cytokines, and chemotherapeutic agents, leading to suboptimal immune response 16. Cuproptosis, a newly identified form of cell death resulting from intracellular copper accumulation, garnering enormous attentions in the field of cancer research because of its high potency in inducing multiple forms of cell death and overcoming drug resistance 17,18. Recent studies demonstrate that cuproptosis inducers trigger mitochondrial DNA (mtDNA) release, activating cGAS-STING pathway and inflammatory caspases, thereby synergizing with ferroptosis to amplify immunogenic cell death 19,20. This synergy is critical for overcoming cold tumor resistance: Cuproptosis-induced DAMPs (ATP/HMGB1) recruit dendritic cells and CD8^+^ T cells, while ferroptosis further enhance tumor antigen presentation and T-cell infiltration, collectively reprogramming immunosuppressive microenvironments 21. Therefore, there is a critical need to explore effective strategies to increase the accumulation of Cu ionophores and inducers such as elesclomol (ES) in tumor tissues, permitting the delivery of Cu into tumor cells and eventually generating multi-mode cell death for immune responses.

To maximize the effectiveness of ICD-based therapies, several factors must be meticulously controlled, including the type, dosage and duration of cell death inducers, as well as their efficient accumulation at tumor sites 22. The co-administration of cell death inducers as simple mixture of soluble formulations cannot guarantee expected therapeutical responses because of their chaotic dissemination *in vivo* that limit immunogenicity and clinical usage by transient responses and various adverse effects. Stimuli-responsive drug delivery systems have emerged as promising platforms for precision cancer treatment, which is crucial for optimizing cancer therapy 23. These advanced delivery systems can facilitate the on-demand release of cell death inducers in tumor, improve cellular internalization and minimize off-target cytotoxicity 24.

Herein, we designed a tumor-targeted nanoinducer based on copper ionophores and pH-sensitive polymers, ES-Cu@PDPA nanoparticles (PEC NPs) for enhanced antitumor immunity. PEC NPs remain as intact form at physiological pH (7.4) during blood circulation and effectively accumulate in tumor tissues with simultaneous release of ES-Cu (EC). EC can be dissociated into ES and Cu^2+^ in the tumor tissue, working in a concerted way to not only kill cancer cells by cuproptosis, but also induce immune responses. Typically, the released Cu^2+^ binds to ferredoxin 1 (FDX1) and reduced to Cu^+^, while ES chelates and transports extracellular Cu^2+^ into tumor cells, leading to mitochondrial stress and the disruption of cellular energy 25,26. Moreover, Cu^2+^ can be reduced to Cu^+^ by endogenous overexpressed glutathione (GSH) and exert Fenton-like activity, promoting ROS generation and amplifying oxidative stress for further triggering PANoptosis with key characteristics of pyroptosis, apoptosis, and necroptosis. Potent ICD effect was induced, allowing to reprogram immunosuppressive TME and ultimately induces effective anti-tumor immune responses in the CT26 colon cancer model. Finally, a synergistic effect was demonstrated when the further combination of PD-1 antibody. Together, this study demonstrated the potential of tumor-specific NPs to stimulate robust and persistent antitumor immunity by mobilizing multimodal cell deaths, helping to guide the rational design of PANoptosis inducers for future cancer immunotherapy.

## Results and Discussion

### Preparation and Characterization of PEC NPs

**Figure 1:**
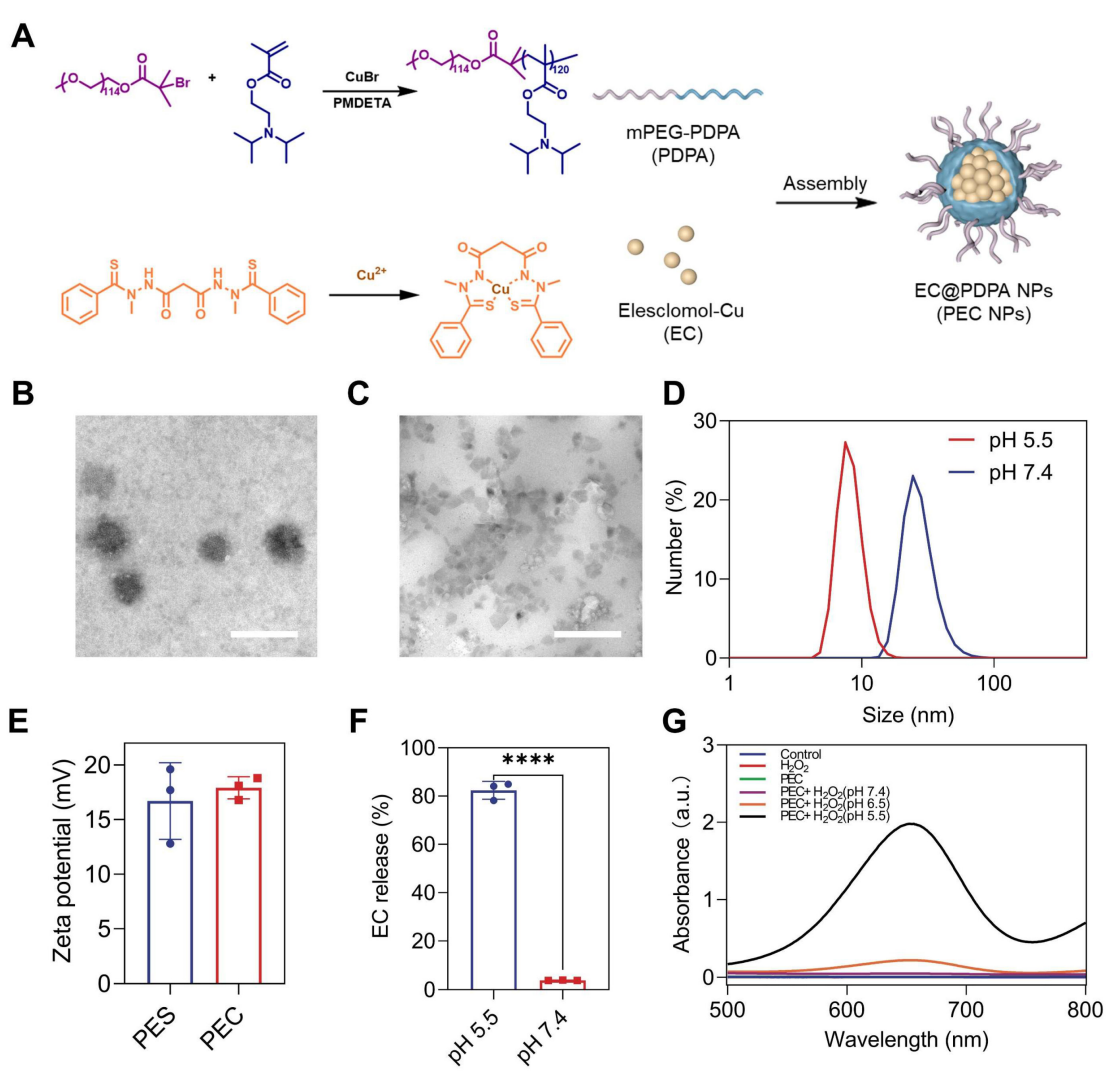
Characterization of PEC NPs. (A) Schematic diagram of nanoparticle synthesis. (B) Representative TEM images of PEC NPs at pH 7.4 and (C) pH 5.5 (Scale bar: 100 nm). (D) Hydrodynamic size of PEC NPs in PBS at pH 7.4 or pH 5.5. (E) Zeta-potential of PES NPs and PEC NPs. (F) Cumulative release of EC from PEC NPs in PBS at pH 7.4 or pH 5.5. (G) Absorption spectrum of ox-TMB in medium with different pH at 2 h. Data are shown as mean ± SD (n = 3 biologically independent experiments per group; two-tailed unpaired Student's t-test). *: p < 0.05, **: p < 0.01, ***: p < 0.001, ****: p < 0.0001.

The ultra pH-sensitive polymer, polyethylene glycol-block-poly (2-diisopropylaminoethyl methacrylate) (PDPA), was selected as a carrier to deliver EC, thereby granting the NPs with stability in neutral conditions and effective release in acidic tumor tissue. The synthesis process of PEC NPs is shown in Figure 1A. The successful synthesis of PDPA was confirmed by ^1^H NMR and Gel permeation chromatography (GPC) (Figure S1, S2 and Table S1). Transmission electron microscopy (TEM) revealed that the NPs exhibited a homogeneous spherical morphology (Figure 1B). Under acidic conditions (pH < 6.0), the NPs dissociated into cationic unimers with precipitated EC (Figure 1C). Dynamic light scattering showed that the diameter PEC NPs of 29.5 nm at pH 7.4. At pH 5.5, the tertiary amines in the hydrophobic block were protonated, resulting in the disassembly of NPs with a diameter of 7.3 nm (Figure 1D). Additionally, the zeta potential of PEC NPs was 17.9 mV (Figure 1E), which is slightly higher compared to PES NPs. The pH-responsive PDPA polymer (pKa = 6.6) enables tumor-targeted nanocarrier disassembly via tertiary amine protonation below pH 6.6, triggering: (i) inter-amine electrostatic repulsion, (ii) hydrophilicity surge, and (iii) osmotic swelling-collectively driving micelle dissociation at tumor acidity (Figure S3). Experimental validation confirms precise pH-dependency. The encapsulation efficiency (EE) of EC for PEC NPs was calculated to be 62.3% ± 5.6%, with a drug loading (DL) of 3.46 ± 0.31%. Moreover, the elemental spectra consisting of Cu, O and S elements further supported the successful encapsulation of EC in PEC NPs (Figure S4). Besides, X-ray photoelectron spectroscopy (XPS) further confirmed that the PEC incorporated Cu, O, N and C elements (Figure S5).

Subsequently, the release of EC from PEC NPs under various conditions was measured. As illustrated in Figure 1F, less than 5% of EC was released from PEC NPs over a 24 h period under neutral conditions (pH 7.4), indicating that PDPA polymers exhibit satisfactory stability in normal physiological conditions. In contrast, the release of EC from PEC NPs was significantly accelerated (≈ 86.7%) under acidic conditions (pH 5.5). These results demonstrate the ultra-pH-sensitive of PDPA polymer and tumor specific EC release. In order to validate the stability of PEC NPs, we have conducted comprehensive stability tests by monitoring the hydrodynamic diameter of PEC NPs in five distinct media (water, PBS, saline, DMEM, and 10% FBS DMEM) under varying temperatures (4 ℃ and 37 ℃) and time intervals (24 h and 7 days). As demonstrated in Figure S6A, PEC NPs maintained their particle size without significant changes across all tested media after 24 h of incubation at both 4 ℃ and 37 ℃. Furthermore, long-term stability was confirmed in Figure S6B, which shows that PEC NPs retained consistent particle sizes for up to 7 days at 4 ℃. These results collectively demonstrate the robust stability of PEC NPs under physiologically relevant conditions.

Next, the peroxidase (POD)-like catalytic activities of PEC NPs were assessed. In the presence of H_2_O_2_, the •OH generated by EC efficiently converted 3,3',5,5'-tetramethylbenzidine dihydrochloride (TMB) to blue oxidized form (oxTMB), showing absorbance peaks at 370 and 652 nm. Importantly, a pH- and time-dependent •OH generation was clearly observed (Figure 1G and Figure S7). At a pH of 5.5, the absorbance of oxTMB is approximately 9.0- and 41.3-fold over that of pH 6.5 and pH 7.4, respectively. The data further demonstrates the specificity and safety profile of PEC NPs, which are capable of inducing POD-like catalytic activity specifically in the acidic TME and within tumor cells, while remaining inert during systemic circulation.

### The Cellular Uptake and Antitumor Activity of PEC NPs *in vitro*

**Figure 2:**
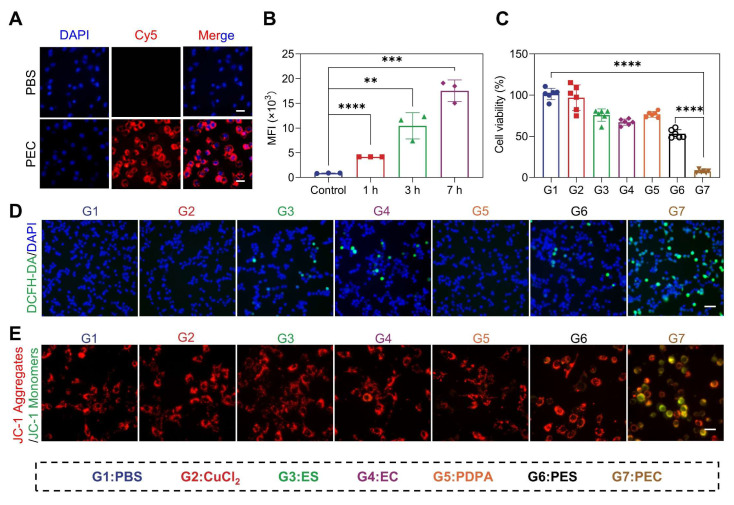
The *in vitro* cytotoxicity of PEC NPs on CT26 cells. (A) Fluorescence microscope images of (Scale bar: 50 μm) and (B) quantitative analysis of intracellular mean fluorescence intensity (MFI) of cells following co-incubation with PEC^Cy5^ NPs. (C) Viability of CT26 cells after incubation with various formulations for 24 h (n=6). (D) Fluorescence microscope images of ROS generation in cells under different treatments (Scale bar: 50 μm). (E) Fluorescence microscope images depicting mitochondrial membrane potential using JC-1 (Scale bar: 50 μm). Data are represented as mean ± SD (n = 3 biologically independent experiments per group). One-way ANOVA followed by Tukey's test in (B) and (C). *: p < 0.05, **: p < 0.01, ***: p < 0.001, ****: p < 0.0001.

Given the pH-responsive release and ROS generation, we next investigated the antitumor efficacy of PEC NPs *in vitro*. The internalization of PEC NPs increased proportionally with incubating time (Figure 2A, 2B, and Figure S8). Further investigation into the endocytosis mechanism revealed that cellular uptake was inhibited by chloroquine and amiloride (Figure S9), suggesting the internalization was occurred via the macropinocytosis and clathrin-mediated endocytosis 27.

Furthermore, the* in vitro* cytotoxicity of PEC NPs against the CT26 cells was evaluated. PDPA polymer exhibited minimal cytotoxicity, while modest cell growth inhibition was shown in cells treated with Cu^2+^, ES, and EC (Figure 2C). As expected, PEC NPs displayed the highest cytotoxicity, significantly higher than that of PES NPs. Compared with unbound ES or pure ES, the enhanced toxicity of PEC NPs is attributed to the significant increasement of intracellular Cu^2+^, thereby accelerating copper-mediated cytotoxicity.

Considering the efficient intracellular delivery of EC and further transportation of Cu^2+^ into mitochondria and cytoplasm, we reasoned that PEC NPs could generate significant amount of ROS and disrupt redox homeostasis. For this, intracellular ROS levels were detected. PEC NPs-treated cells displayed the strongest green fluorescence, indicating extensive ROS production (Figure 2D). Furthermore, we evaluated the effect of ROS on mitochondrial damage. As shown in Figure 2E, PEC NPs-treated cells displayed the brightest green fluorescence and the weakest red fluorescence, indicating superior mitochondrial damage. In contrast, other treatments did not induce significant mitochondrial damage, as evidenced by strong red fluorescence and weak green fluorescence. These results suggest that PEC NPs induce severe mitochondrial damage and generate extensive ROS to effectively kill tumor cells.

### PEC NPs Effectively Induce Cuproptosis and PANoptosis

**Figure 3:**
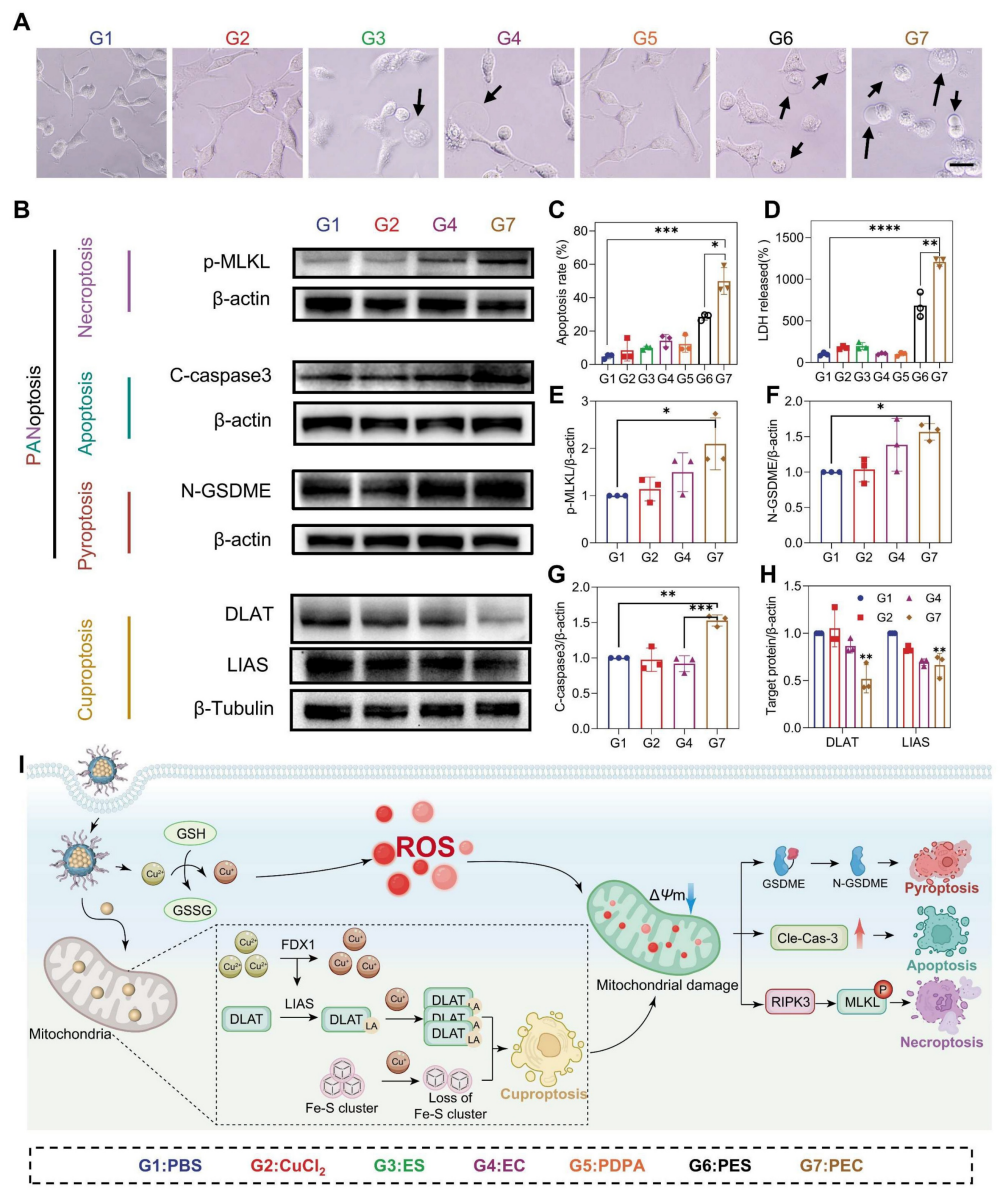
Cuproptosis and PANoptosis induction by PEC NPs. (A) Representative morphological changes of CT26 cells following various treatments (Scale bar: 50 μm). (B) Western blot analysis of cleaved caspase-3, N-GSDME, p-MLKL, LIAS and DLAT expression in cells under various treatments. (C) Quantitative analysis of the apoptosis rate in CT26 cells following various treatments. (D) LDH release from cells following various treatments. (E-H) relative protein expression levels (target protein/loading control) of cleaved caspase-3, N-GSDME, p-MLKL, LIAS and DLAT. (I) Schematic representation of immune response induced by PEC NPs. Data are represented as mean ± SD (n = 3 biologically independent experiments per group). One-way ANOVA followed by Tukey's test in (B-H). *: p < 0.05, **: p < 0.01, ***: p < 0.001, ****: p < 0.0001.

The remarkable efficacy prompted further investigation into the mechanisms of cell death. Cuproptosis, a form of cell death resulting from intracellular copper accumulation and proteotoxic stress 25. Key markers of cuproptosis, including DLAT and LIAS proteins were examined. The destabilization of LIAS and abnormal oligomerization of DLAT was clearly shown within cells treated with PEC NPs, suggesting its capability in inducing cuproptosis (Figure 3B and 3H). In the meantime, EC alone produced comparable results, though to a lesser extent than observed in the PEC NPs group. Expectedly, the pretreatment with copper-chelating agent (tetrathiomolybdate, TTM) dramatically reduced the cytotoxicity of PEC NPs (Figure S10). These data demonstrated that efficient cuproptosis was triggered by PEC NPs.

ROS can trigger pyroptosis in multiple cancers, a form of inflammatory cell death characterized by cell membrane perforation, swelling, and release of contents like lactate dehydrogenase (LDH) 28. To test whether PEC NPs induce pyroptosis, we examined CT26 cell morphology after various treatments. PEC NPs-treated cells exhibited membrane swelling and giant blebbing (indicated by black arrows), characteristic of pyroptosis (Figure 3A). However, cells with other treatments showed less or minor degree of membrane bubbles. Furthermore, PEC NPs induced the highest levels of LDH release, followed by PES NPs, with minimal release in other groups (Figure 3D). The LDH release in the PEC group was 12.1-fold higher than PBS and 1.8-fold higher than PES, indicating strong pyroptosis induction by PEC NPs. In classical pyroptosis, inflammatory caspase-3 is activated to cleave gasdermin E (GSDME), thereby generating N-terminal fragments (N-GSDME) that perforate the cell membrane. As shown in Figure 3B and 3F, an increased expression of cleaved N-GSDME was noted in the PEC group, indicating the induction of pyroptosis via the caspase-3/GSDME pathway.

Membrane bubbling compromises cellular integrity, leading to increased permeability and apoptosis. Therefore, the apoptotic rate of CT26 cells treated with PEC NPs was examined (Figure 3C and Figure S11). EC alone increased the total apoptotic rate from 4.8% to 14.2%, while PDPA-assisted delivery further raised it to 49.9%. Additionally, the PEC group showed upregulated cleaved caspase-3 (Figure 3B and 3G), indicating efficient induction of apoptosis.

As the close correlation of membrane rupture and necroptosis, we investigated the possibility of necroptosis induced by PEC NPs. Necroptosis is a programmed form of cell death mediated by receptor-interacting protein kinase 1/3 (RIPK1/3) and mixed lineage kinase domain-like protein (MLKL) 29. Interestingly, the PEC group showed a significant increase in the expression of p-MLKL (Figure 3B and 3E), demonstrating the activation of necroptosis. Mechanistically, after being endocytosed into cancer cells, PEC NPs was activated in an acidic lysosome environment, generating abundant ROS. Subsequently, the released EC was transferred to mitochondria along with enrichment of Cu^2+^ (Figure 3I). Mitochondrial dysfunction not only induces the aggregation of toxic proteins that cause cuproptosis, but also triggers the accumulation of ROS. Simultaneously, cuproptosis enhanced by concertedly disrupt mitochondrial metabolism, further exacerbating intracellular oxidative stress and resulting in PANoptosis.

These data collectively indicate that PEC NPs activates cuproptosis and achieves PANoptosis. As the sensitivity of tumor cells to death mechanisms vary significantly, attributed to the absence or silence of key genes that linked to RCD pathways, which complicated the tumor killing effect and further immune activation. For instance, GSDME, a key molecule in pyroptosis, is frequently downregulated or silenced. Similarly, RIPK3, involved in necroptosis, is often downregulated 26. Therefore, the induction of PANoptosis by PEC NPs triggered robust tumor killing through multiple death mechanisms and subsequent immune activation, which is especially valuable when tumor cells are resistant to specific treatment that target single death pathway.

### PEC NPs Triggered Potent ICD

**Figure 4:**
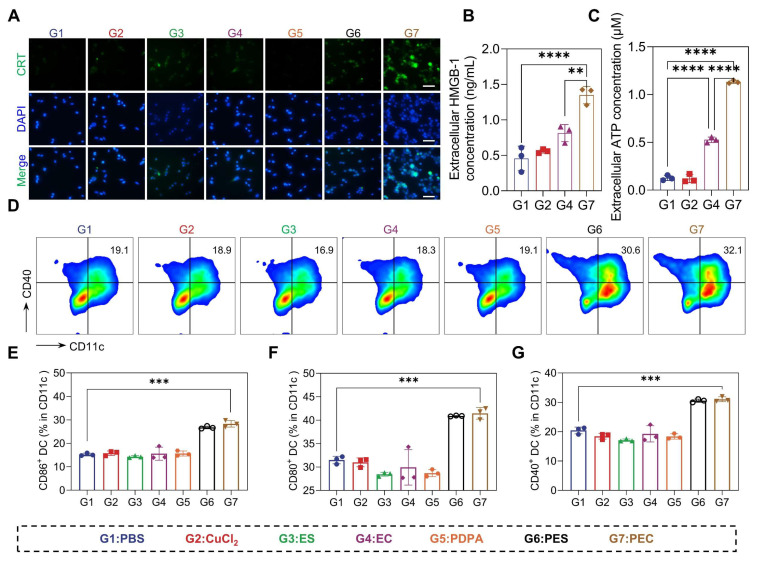
* In vitro* ICD induced by PEC NPs. (A) Fluorescence microscope images showing the translocation of CRT to the surface of CT26 cells upon exposure to PEC NPs (Scale bar: 50 μm). Extracellular HMGB-1(B) and ATP(C) levels of CT26 cells after under different treatments. (D) Flow cytometry analysis of BMDCs maturation after incubation with CT26 cells under various treatments. (E-G) Quantitative analysis of costimulatory molecules CD40, CD80, and CD86 on BMDCs. Data are represented as mean ± SD (n = 3 biologically independent experiments per group). One-way ANOVA followed by Tukey's test in (B), (C), (E), (F), (G). *: p < 0.05, **: p < 0.01, ***: p < 0.001, ****: p < 0.0001.

Given the capability of PEC NPs in the induction of cuproptosis and subsequent ICD, we thus hypothesized effective immune activation could be triggered 30-32. However, there was no significant change in either the CuCl_2_ and EC groups. Moreover, the brightest green fluorescence indicative of CRT is localized around the cell membrane in cells treated with PEC NPs, whereas faint fluorescence is observed in other experimental groups (Figure 4A). Significant release of ATP and HMGB-1 secretion was observed in CT26 cells treated with PEC NPs (Figure 4B, C and Figure S12). DAMPs released during ICD bind to pattern recognition receptors of dendritic cells (DCs), activating innate and adaptive immunity.

The immunogenicity of PEC NPs was further evaluated using an *in vitro* DCs maturation assay. PEC NPs and PES NPs significantly upregulated the expression of co-stimulatory molecules compared to other groups, indicating that the ICD effect of tumor cells induced DC maturation (Figure 4D-G). These data collectively suggest that PEC NPs effectively evoked ICD effect, thereby eliciting a robust immune response.

### The Antitumor Efficacy of PEC NPs *In Vivo*

**Figure 5:**
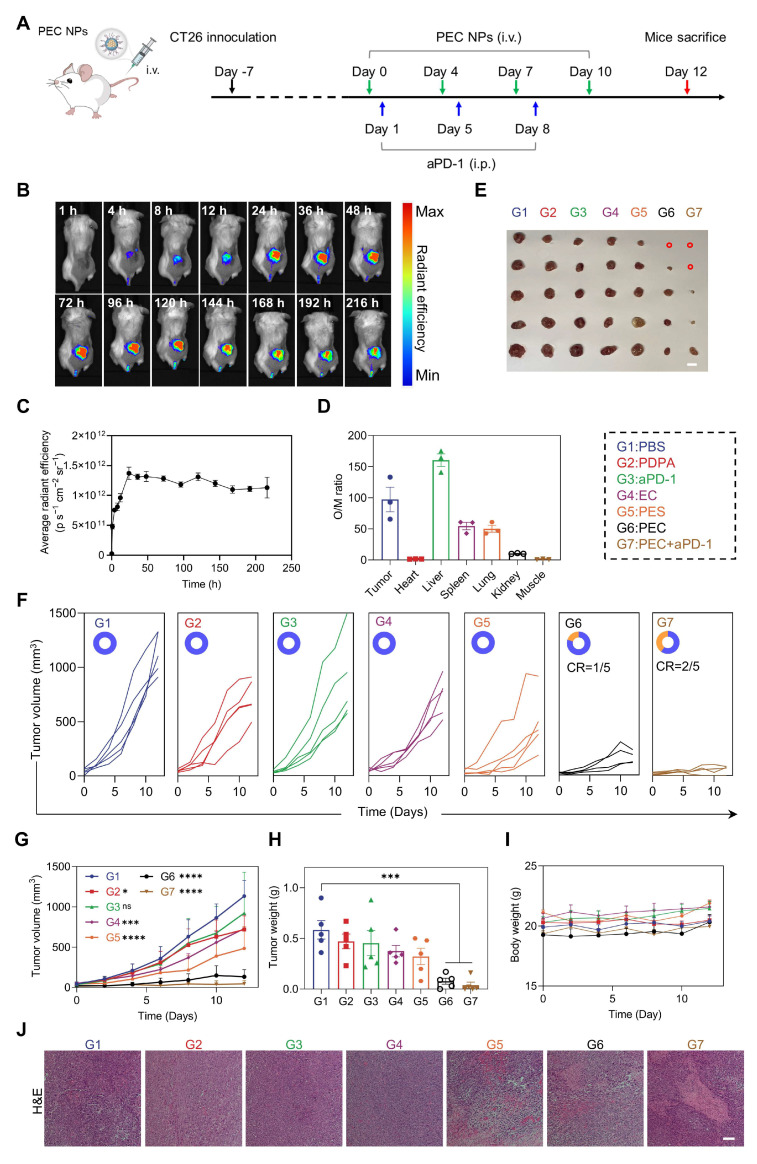
Assessment of *in vivo* anti-tumor efficacy of PEC NPs. (A) Schematic illustration of the experimental schedule for CT26 tumor-bearing mice. (B) *In vivo* fluorescence imaging of PEC NPs. (C) Pharmacokinetics analysis of PEC NPs. (D) Organ to muscle ratios (O/M ratio) at 24 h post-injection of PEC NPs. (E) Corresponding tumor photographs (Scale bar: 1 cm), (F) individual tumor volume curve (CR, complete regression), (G) tumor growth curves, (H) *ex vivo* tumor weights and (I) body weight changes of mice of the CT26 tumor-bearing mice after various treatments. (J) H&E staining of the tumor tissues (Scale bar: 250 μm). Data are represented as mean ± SEM (n = 3 biologically independent mice per group imaging experiments and n = 5 biologically independent mice per group in antitumor efficacy study). Two-way ANOVA in (G), One-way ANOVA followed by Dunnett's test in (H), *: p < 0.05, **: p < 0.01, ***: p < 0.001, ****: p < 0.0001.

After demonstrating the significant antitumor activity of PEC NPs *in vitro*, we further evaluated their therapeutic efficacy *in vivo*. Hemocompatibility assessment revealed exceptional blood compatibility of PEC NPs, with hemolysis rates < 5% across therapeutic concentrations (Figure S13). The tumor accumulation of PEC^Cy5^ NPs was observed using an *in vivo* imaging system (Figure 5B). The tumor-targeting kinetics of PEC NPs exhibit a triphasic profile: Early accumulation (1-12 h post-injection) features rapid extravasation into tumors. Peak enrichment occurs at 24 h driven by EPR effect and pH ultra-sensitive. Long-term retention (72-216 h) demonstrates exceptional intratumoral persistence (Figure 5C). Subsequently, dominant tumor targeting was quantitatively confirmed by Organ-to-Muscle Fluorescence Ratio (O/M Ratio) analysis, revealing a O/M Ratio of 97.2 in tumor, indicating efficient tumor distribution of PEC NPs (Figure S14 and Figure 5D).

Next, we assessed the* in vivo* antitumor efficacy of PEC NPs. Mice were randomly assigned to different treatment groups (Figure 5A). EC alone exhibited limited antitumor activity, with an inhibition rate of 36.0%, primarily due to inefficient tumor delivery (Figure 5E-H). In contrast, PEC NPs demonstrated superior antitumor activity, with an inhibition rate of 88.3%. The combination of PEC NPs with aPD-1 further enhanced therapeutic efficacy, achieving an inhibition rate of 96.0% and 40.0% of mice with tumor free. PES NPs showed modest antitumor activity with an inhibition rate of 44.8%, which was higher than that of EC alone, supporting the notion that PDPA improved the water solubility and targeting efficiency of EC. As expected, both PEC and PEC + aPD-1 treatments significantly prolonged the survival period (Figure S15). We further investigated whether PEC NPs could induce cuproptosis *in vivo*. Consistent with the *in vitro* findings, a significant decrease in LIAS expression was observed in tumor tissues treated with PEC NPs, PES NPs, and PEC NPs + aPD-1 (Figure S16). These data suggest that PEC NPs can induce cuproptosis in tumor cells.

The body weight of the mice showed no statistically significant differences among the groups. (Figure 5I). Additionally, histological examination of the major organ tissues and tumor sections was performed using H&E staining. Pronounced nuclear crumpling and tissue damage were observed in the PEC group (Figure 5J), whereas no significant abnormalities were detected in the major organ tissues (Figure S17). Collectively, these data indicate that PEC NPs induce potent antitumor activity while demonstrating excellent biocompatibility.

### *In Vivo* Anti-Tumor Immune Responses Analysis

**Figure 6:**
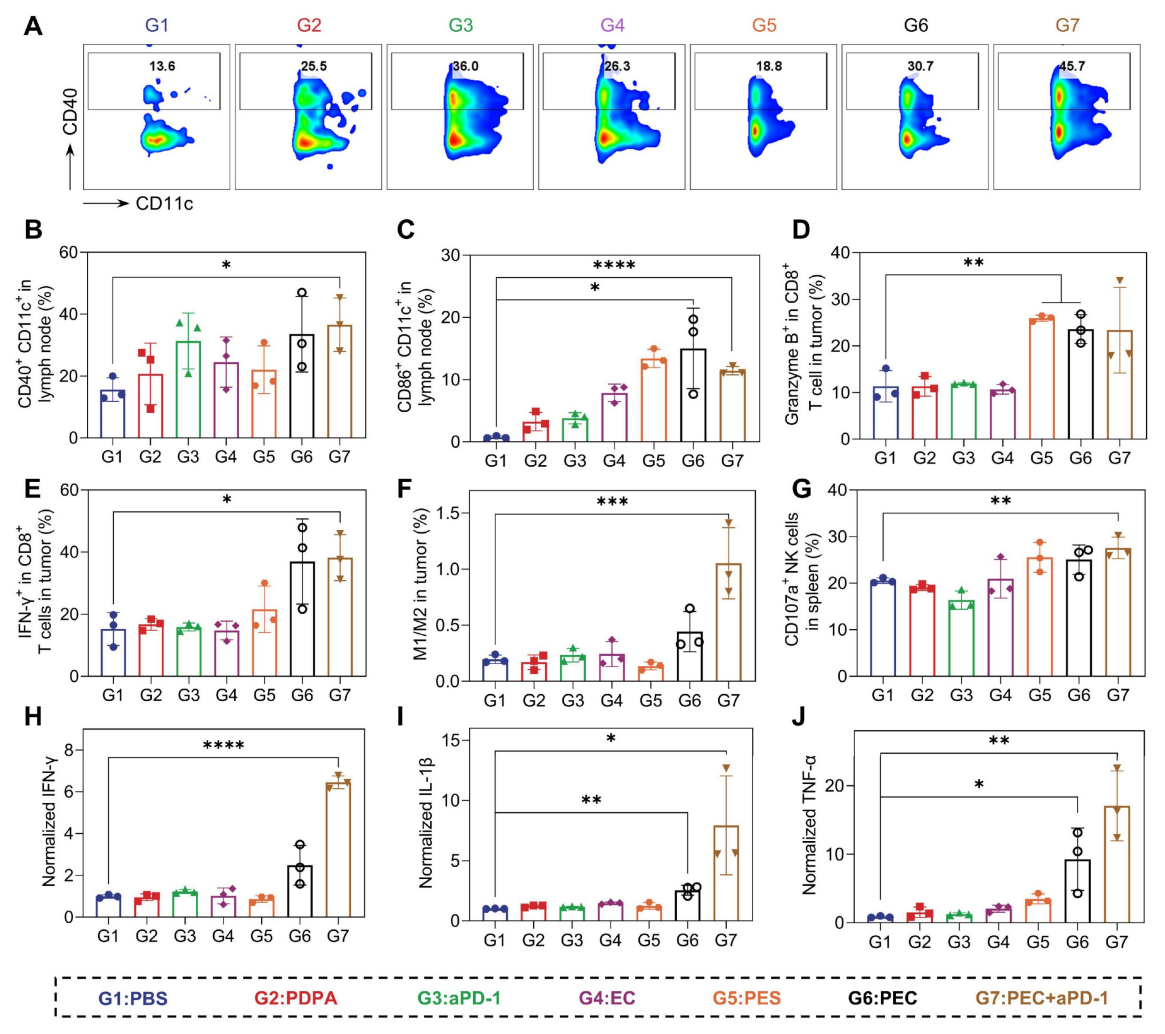
PEC NPs activate potent antitumor immune responses *in vivo*. (A) Flow cytometry analysis of CD40^+^CD11c^+^ cells in lymph node after different treatments. Histogram analysis of the percentages of CD40^+^CD11c^+^ cells(B) and CD86^+^CD11c^+^ cells(C) in lymph node. (D) The ratio of tumor-infiltrating Granzyme B^+^ in CD8^+^ T cells. (E) The ratio of tumor-infiltrating IFN-γ^+^ in CD8^+^ T cells. (F) The ratio of M1-type macrophage/M2-type macrophage in tumor. (G) Histogram analysis of the percentages of CD107^+^ in NK cells. (H-J) The secretion of cytokines IFN-γ, IL-1β and TNF-α in tumor tissue. Data are represented as mean ± SD (n = 3 biologically independent experiments per group). One-way ANOVA followed by Dunnett's test in (B), (C), (D), (E), (F), (H), (I), (J) and student's t-test in (G), *: p < 0.05, **: p < 0.01, ***: p < 0.001, ****: p < 0.0001.

We next explore the mechanism of *in vivo* therapeutical response. Firstly, the maturation of DCs in tumor-draining lymph nodes were analyzed. 33.5% (CD40^+^CD11c^+^) and 15.0% (CD86^+^CD11c^+^) of DCs were matured in mice treated with PEC NPs (Figure 6A-C), indicating effective DC maturation and allow the activation of T cell. Subsequently, the infiltration of T cells in tumor tissues was analyzed. The tumor-killing ability of CD8⁺ T cells relies on cell-lytic enzymes and cytokines. In the PEC and combination therapy groups, significant increases in IFN-γ and granzyme B from CD8^+^ T cells were observed (Figure 6D and E). These findings suggest that PEC NPs effectively promotes the maturation and proliferation of T cells* in vivo*. Natural killer (NK) cells, play a crucial role in anti-tumor, antiviral and immunoregulatory functions 33. The combination of aPD-1 and PEC NPs synergistically enhanced the proportion of activated NK cells in the spleen (Figure 6G), indicating that their cooperative action potentiates NK cell proliferation and activation. Tumor-associated macrophages (TAMs) are reported to account for a large proportion of tumor mass 34. TAMs are mainly classified into pro-inflammatory M1-type (CD80^+^CD206^-^) and anti-inflammatory M2-type (CD80^-^CD206^+^). In tumor tissues, M2-TAMs are widely distributed and inhibit the proliferation of T cells and induce tumor immune evasion. As shown in Figure 6F, the M1/M2 ratio in the PEC + aPD-1 group showed a significant increase compared with the other groups, and a moderate elevation was also noted in the PEC group. The data suggest that PEC NPs induced the repolarization of TAMs from M2 to M1 macrophages, helping to relieve the suppressive TME for cascade immune response.

Furthermore, changes in cytokine were investigated using an enzyme-linked immunosorbent assay (ELISA). PEC NPs induced higher levels of intratumoral cytokines IFN-γ, TNF-α and IL-1β, and the combination with aPD-1 further enhanced the secretion levels, suggesting that IFN-I and pro-inflammatory cytokines mediate an effective anti-tumor immune response to induce tumor regression (Figure 6H-J). Taken together, PEC NPs effectively activated the immune system and reversed the immunosuppressive TME, leading to potent tumor growth inhibition.

## Conclusion

TME is characterized by significant heterogeneity and complexity, often resulting in resistance to single-mode RCD and suboptimal immune activation. In this study, we report the development of TME-responsive Cu ionophore-based nanomaterials designed to induce cuproptosis and achieve subsequent PANoptosis, thereby enhancing ICD for cancer immunotherapy. For this, a pH-sensitive polymer was synthesized to encapsulate both ES and Cu^2+^ into nanoparticles, specifically PEC NPs. These PEC NPs exhibit excellent structural integrity and prolonged *in vivo* circulation. Upon exposure to the acidic conditions prevalent in the TME, the PEC NPs rapidly dissociated to release Cu^2+^ and ES. The PEC NPs facilitate the uptake of EC complexes by tumor cells, leading to a significant increase in intracellular Cu^2+^ levels. This elevation in Cu^2+^ concentration disrupts cellular homeostasis, thereby initiating multiple forms of RCD. As a result, robust ICD effects are induced, releasing substantial amounts of DAMPs that effectively activate the immune system. Moreover, PEC NPs alleviate TME-associated immunosuppression by significantly increasing the M1/M2 macrophage ratio. This shift creates an immune-supportive TME that stimulates a cascade of immune responses. In a colon cancer model, these NPs demonstrate potent tumor growth inhibition and survival time prolongation. Notably, a synergistic effect was shown when further combined with immune checkpoint blockade therapy. Overall, this study provides a tumor-responsive nanomedicine that induce multimodal RCDs via cuproptosis, addressing key challenges associated with single ICD therapies, including limited immune responses and considerable variability in sensitivity to different death modalities.

## Supplementary Material

Supplementary materials and methods, figures and table.

## Figures and Tables

**Scheme 1:**
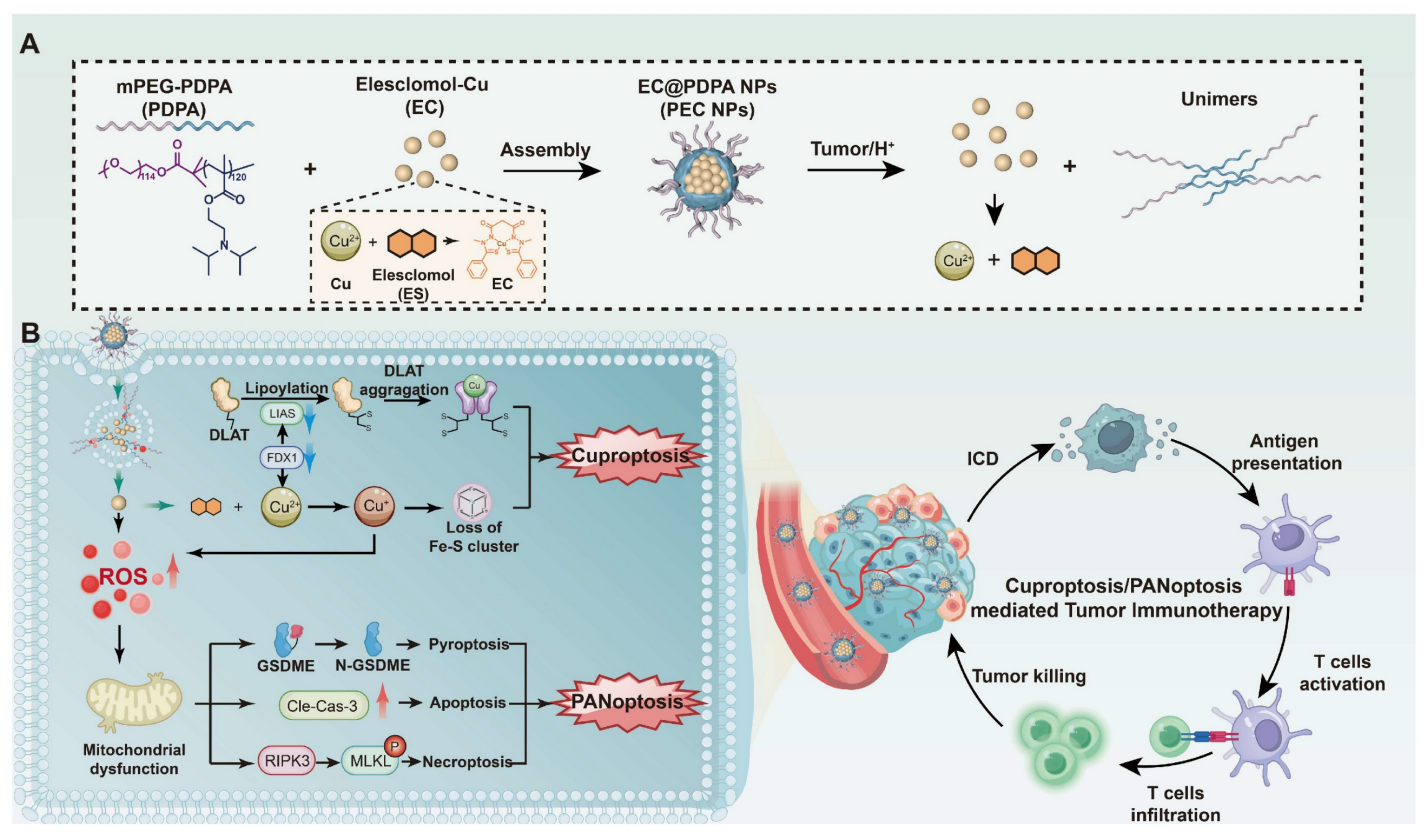
Illustration of synergistic cell death induced by PEC NPs for enhancing antitumor immunotherapy.
