# Supplementary material for: Tumor-responsive cuproptosis nanoinducer realizing efficient PANoptosis for enhanced cancer immunotherapy
Source: Theranostics. 2025 Aug 16;15(17):9294–305. doi: 10.7150/thno.115275 (PMC12439466; doi:10.7150/thno.115275)
Supplement: Supplementary file 1 — Supplementary materials and methods, figures and table. [file thnov15p9294s1.pdf]

Supporting Information

Tumor-Responsive Cuproptosis Nanoinducer Realizing Efficient PANoptosis for Enhanced Cancer Immunotherapy

Kaiqing Yun[a,c,‡], *Xiaohong Yu*[b, ‡], *Shuang Liang*[a,c], *Qingling Wang*[f], *Ziyi Zhang*[e], *Yue Han*[f], *Yueyang Zhao*[g], *Yuxuan Peng*[a,c], *Lang Rao*[d, *], *Yong Cui*[b,*] *and Zhaohui Wang*[a,c,*]

‡These authors contributed equally.

[a]State Key Laboratory of Bioactive Substance and Function of Natural Medicines, Institute of Materia Medica, Chinese Academy of Medical Sciences & Peking Union Medical College, Beijing 100050, China.

[b]College of Medical Devices, Shenyang Pharmaceutical University, Shenyang 110016, China

[c]Beijing Key Laboratory of Drug Delivery Technology and Novel Formulation, Institute of Materia Medica, Chinese Academy of Medical Sciences & Peking Union Medical College, Beijing 100050, China.

[d]Institute of Chemical Biology, Shenzhen Bay Laboratory, Shenzhen 518132, China.

[e]School of Life Sciences and Biopharmaceutical Science, Shenyang Pharmaceutical University, Shenyang 110016, China.

[f]Wuya College of Innovation, Shenyang Pharmaceutical University, Shenyang 110016, China.

[g]Hebei Key Laboratory of Innovative Drug Research and Evaluation, School of Pharmaceutical Sciences, Hebei Medical University, Shijiazhuang 050017, China.

Materials

All reagents were purchased from commercial sources or synthesized and used without further purification unless specified. Copper(II) chloride dihydrate ($CuCl_2 \cdot 2H_2O$, Sigma-Aldrich), Elesclomol (ES, Bidepharm), 2-dipropylaminoethyl methacrylate (DPA-MA, Sigma-Aldrich), 2-aminoethyl methacrylate hydrochloride(AMA-MA, Adamas), 2',7'-dichlorofluorescein diacetate (DCFH-DA, Beyotime), tetrathiomolybdate(TTM, MCE), N, N, N', N'', N''-pentamethyldiethylenetriamine (PMDETA, Adamas), RPMI 1640 with L-Glutamine (M&C Gene Technology), Mitochondrial membrane potential assay kit with JC-1 (Beyotime, C2006), LDH Cytotoxicity Assay Kit (Beyotime, C0017), Annexin V-FITC/PI Apoptosis Detection Kit (meilunbio, MA0220-2), Enhanced BCA Protein Assay Kit (Beyotime, P0009), caspase-3 (abcam, ab184787), N-terminal-GSDME (abcam, ab222407), Phospho-MLKL (abcam, ab196436), FDX1 (immunoway, YT18131), LIAS (immunoway, YT8133), DLAT (immunoway, Y1328), HMGB-1 (abcam, ab18256), β-tubulin (proteintech, 10068-1-AP). All antibodies used for flow cytometry were purchased from Invitrogen.

Methods

Synthesis of mPEG-*b*-P(DPA-*r*-AMA) (PDPA)

PDPA was synthesized using atom transfer radical polymerization (ATRP) method. Initially, methoxypolyethylene glycol bromide ($mPEG_{5k}$-Br) (250 mg, 0.05 mmol) was placed in a schlenk flask and dissolved in DMF (1 mL) under brief heating. Subsequently, the monomers 2-dipropylaminoethyl methacrylate (DPA-MA) (460 mg, 8 mmol) and 2-aminoethyl methacrylate hydrochloride (AMA-MA) (25 mg, 0.15 mmol), along with the ligand N, N, N', N'', N''-pentamethyldiethylenetriamine (PMDETA) (11 μL, 0.055 mmol) were added. Isopropanol (1 mL) was then introduced to the mixture. The solution underwent three freeze-pump-thaw cycles to remove oxygen. Copper(I) bromide (CuBr) (7.8 mg, 0.055 mmol) was added to the flask under a nitrogen atmosphere, and the flask was sealed under vacuum. Polymerization was

conducted at 42 °C overnight. The reaction mixture was diluted with THF and passed through a neutral alumina (Al_2O_3) column to remove the catalyst. THF was removed by rotary evaporation, and the residue was dialyzed using 100 kDa MWCO dialysis bag in distilled water for 24 h. The product was lyophilized to obtain a white solid, which was stored at -80 °C.

For further fluorescence labeling, polymers (6-8 mg, 0.33 mmol) were dissolved in DMF at a concentration of 10 mg/mL. Cy5 NHS ester was dissolved in 100 μL of DMF and then added to the reaction vial. The reaction mixture was adjusted to 1 mL with excess methanol and incubated at room temperature for 24 h with stirring at 500 rpm. The purification of product was carried out by ultrafiltration method with a cutting-off MWCO 10 KDa.

Preparation and characterization of PEC NPs

The mixture of PDPA (10 mg/mL in anhydrous ethanol) and ES (10 mg/mL in DMSO) was supplemented with $CuCl_2$ (10 mg/mL in DMSO), then sonicated in 10 mL of water using an ultrasound probe. Organic solvents and unencapsulated reactants were subsequently removed by ultrafiltration (MWCO: 100 kDa). The resulting PEC was adjusted to a volume of 1 mL and stored at 4 °C. Using PDPA and ES as starting materials, PES NPs was synthesized using the similar method. The morphology of PEC NPs was analyzed using transmission electron microscopy, following negative staining with uranyl acetate. The particle size and zeta potential of PEC and PES were characterized using dynamic light scattering (DLS). UV-Vis spectroscopy was utilized for the quantitative analysis of EC.

To assess the release profile of EC from PEC, NPs were placed in a dialysis bag (MWCO: 100 kDa) and transferred in PBS medium with pH at 5.5 and 7.4, respectively. The release medium was collected at predetermined intervals, and the concentration of released EC was determined using UV–Vis spectrophotometer to calculate the cumulative release percentage.

Stability Assessment of PEC NPs

The stability of PEC NPs were systematically evaluated by monitoring hydrodynamic diameter changes in physiologically relevant media. PEC NPs were dispersed in five distinct media: Deionized water, Phosphate-buffered saline (PBS, pH 7.4), Saline (0.9% NaCl), Dulbecco's Modified Eagle Medium (DMEM), and DMEM supplemented with 10% fetal bovine serum (FBS). Samples were incubated at controlled temperatures of 4 ℃ (refrigerated storage) and 37 ℃ (physiological temperature) for 24 h (short-term stability) and 7 days (long-term stability). Hydrodynamic diameters were measured at predetermined time points using dynamic light scattering (DLS) on a Malvern Zetasizer Nano ZS (Malvern Panalytical, UK). Three independent replicates were performed for each condition.

Detection of Hydroxyl Radicals (•OH)

The generation of hydroxyl radicals(•OH) was investigated using TMB as a probe. A mixture comprising 0.16 mL of PEC micelle solution, 0.02 mL of hydrogen peroxide (H_2O_2, 20 mM), and 0.04 mL of TMB (20 mM) was diluted to a total volume of 0.6 mL with PBS at pH 5.5 and 7.4. Absorption spectra of the solutions were recorded at predetermined intervals using UV-visible spectroscopy.

Cell Lines and Animals

The CT-26 cell lines were obtained from the Institute of Basic Medical Sciences, Chinese Academy of Medical Sciences. CT-26 cells were cultured in RPMI 1640 medium supplemented with 10% fetal bovine serum and antibiotics (penicillin 100 U/mL and streptomycin 100 mg/mL).

Female BALB/c mice (18-20 g) were procured from GemPharmatech Co. Ltd. A total of 1.5×10^6 CT-26 cells were subcutaneously injected into the right flank of the BALB/c mice to establish a colorectal cancer model. All animal experiments were

approved by the Institutional Animal Care and Use Committee of Peking Union Medical College.

Cellular uptake and uptake mechanisms

The cellular uptake of PEC^{Cy5} was analyzed using flow cytometry and fluorescence microscopy. Typically, CT-26 cells were seeded in 24-well plates at a density of 10^4 cells per well and cultured overnight. PEC^{Cy5} was added to the wells, with untreated cells serving as negative controls, and cells were cultured in complete medium. Cells were harvested at 1 h, 4 h and 7 h for fluorescence quantification using flow cytometry. For fluorescence microscopy observation, CT-26 cells were seeded in 24-well plates at a density of 10^4 cells per well and cultured overnight. Three different concentrations of PEC^{Cy5} NPs were incubated with cells for 2 h. After removing the medium, cells were washed three times with PBS, stained nucleus with Hoechst 33342, and images were captured using a fluorescence microscope.

CT26 cells were seeded in 96-well plates (10^5 cells per well) 24 h before treatments. For endocytosis pathway study, a variety of inhibitors including amiloride (20 μM), chlorpromazine (20 μM), genistein (100 μM), cytochalasin D (0.5 μM), chloroquine (20 μM) and methyl-β-cyclodextrin (5 mM) dissolved in serum-containing cell culture media were added to CT26 cells, respectively. The cells were then incubated at 37 °C for 1 h and washed thoroughly with PBS three times. PEC^{Cy5} NPs was then added to the cells. After 4 h incubation at 37°C, the media were aspirated, and the cells were washed with PBS three times and collected after trypsinization. The cellular uptake of PEC^{Cy5} NPs in CT26 was assayed by flow cytometry to quantify.

Cell viability assays

The therapeutic efficacy of nanoparticles was evaluated via CCK-8 assay using CT-26 cells. Cells were seeded at 10^4 cells/well in 96-well plates and cultured overnight. Two independent experimental sets were conducted: First, seven treatment groups (PBS

control, 10 μM $CuCl_2$, ES, EC, PDPA, PES, and PEC) were compared; Second, PEC were tested across concentrations (12.5-200 μM EC equivalent concentration) with or without 10 μM tetrathiomolybdate (TTM). All groups except PBS control underwent 10-minute preconditioning with 10 μM $CuCl_2$ prior to 24 h incubation with respective test materials. Post-treatment, 10% v/v CCK-8 reagent was added to each well and incubated for 1 h before measuring absorbance at 450 nm using a BioTek Synergy H1 microplate reader.

Detection of mitochondrial membrane potential

The JC-1 mitochondrial membrane potential detection kit was employed to assess the alterations in mitochondrial membrane potential. CT-26 cells were seeded at a density of 10^5 cells/well in a 24-well plate and incubated overnight. Cells in the experimental groups, excluding the PBS group, were preincubated with 10 μM $CuCl_2$ in serum-free medium for 10 min. The control group was incubated with 10 μM $CuCl_2$ in serum-free medium, followed by the addition of ES, EC, PDPA and PES. The concentration of ES was 200 nM, and PDPA levels were consistent across all groups. After 4 h incubation, the culture medium was removed, and the cells were washed three times with PBS. Subsequently, the cells were incubated with the JC-1 reagent in the dark at 37 °C for 30 min. Unbound dyes were washed off, and the cells were immediately analyzed using a fluorescence microscope.

Apoptosis analysis

Cell apoptosis was assessed using the Annexin V-FITC apoptosis detection kit. In brief, CT-26 cells were seeded in 24-well plates at a density of 10^5 cells per well and cultured overnight. Prior to the addition of drugs, cells in all experimental groups, except the PBS group, were incubated in serum-free medium containing 10 μM $CuCl_2$ for 10 min. Subsequently, ES, EC, PDPA, PES and PEC (with an ES concentration of 200 nM) were added and incubated for 4 h. The cells incubated only with serum-free medium

containing 10 μM $CuCl_2$ served as the control group. Cells were then washed with PBS for apoptosis detection using the Annexin/PI kit.

Detection of reactive oxygen species (ROS) production

Using 2',7'-dichlorodihydrofluorescein diacetate (DCFH-DA) as a fluorescent probe, we investigated the level of intracellular reactive oxygen species (ROS). Briefly, CT-26 cells were seeded at a density of 10^4 cells per well in 24-well plates and cultured overnight. All experimental group wells, except the control, were incubated with 10 μM $CuCl_2$ in the medium for 30 min before drug addition. The cells incubated with 10 μM $CuCl_2$ medium were designated as the $CuCl_2$ group. Subsequently, ES, EC, PDPA, PES and PEC were added. After incubation for 7 h with 200 nM ES and consistent PDPA in each group, the cell culture medium was replaced with serum-free medium.

The cells were incubated with the ROS probe DCFH-DA (10 μM) for 30 min, washed with PBS, and stained with DAPI to visualize the nucleus. Images were captured using fluorescence microscopy. Further detection and quantification of intracellular ROS levels using DCFH-DA were performed via flow cytometry, as previously described.

LDH levels assay

Lactate dehydrogenase (LDH) release from cells was quantified using an LDH assay kit. Briefly, CT-26 cells were seeded at a density of 5,000 cells per well in 96-well plates and cultured overnight. Before adding the drugs, all experimental groups, except for the PBS group, were incubated in serum-free medium containing 10 μM $CuCl_2$ for 10 min. Following this, ES, EC, PDPA, PES and PEC (with an ES concentration of 200 nM) were added, and the cells were incubated for 10 h. LDH release was then assessed using the LDH assay kit.

Western Blotting (WB) analysis

Briefly, CT26 cells were seeded into 6 wells plate and treated with different

formulations overnight. RIPA cell lysate was used to extract the protein of cells, and the protein concentration was measured by BCA protein assay kit. Then, the loading buffer was added and heated for protein denaturation. The samples were added to SDS-PAGE (sample loading amount is 30 μg), and the proteins were transferred into the PVDF membrane via the gel electrophoresis apparatus (Bio Rad mini, USA). The PVDF membranes were blocked and successively incubated with primary antibodies (including cleaved-caspase-3, N-terminal-GSDME, p-MLKL, DLAT, LIAS, β-actin and β-tubulin) and HRP-labeled secondary antibodies. The images were obtained by gel imaging system after culturing with enhanced chemiluminescent reagent.

Maturation and activation of BMDCs

Bone marrow-derived dendritic cells (BMDCs) were derived from the femur and tibia of mice and cultured in RPMI 1640 medium containing granulocyte-macrophage colony-stimulating factor (GM-CSF, 1000 U/mL) and interleukin-4 (IL-4, 1000 U/mL). The culture medium was refreshed every other day, and cells were harvested on day 7.

CT-26 cells were inoculated at a density of 10^5 cells/well in a 24-well plate and cultured overnight. All experimental group wells, excluding the PBS group, were treated with 10 μM $CuCl_2$ in serum-free medium for 10 min prior to drug addition. The control group was treated with cells and 10 μM $CuCl_2$ in serum-free medium. Subsequently, ES, EC and PDPA were added to the PES and PEC wells and incubated for 2 h and collect the supernatant. After overnight incubation in complete culture medium and removal of the supernatant, BMDCs were inoculated at a density of 5×10^5 cells/well into a 24-well plate, supplemented with the collected tumor supernatant, and cultured for 24 h. The cells were then stained with anti-CD80, anti-CD86, and anti-CD11c antibodies, and flow cytometry was performed to assess the expression of costimulatory molecules CD80 and CD86.

Hemolysis Rate Assay

Fresh mice whole blood anticoagulated with EDTA was centrifuged at 1,500 × g for 10 min to isolate erythrocytes. The collected red blood cells (RBCs) were washed three times with sterile phosphate-buffered saline (PBS, pH 7.4) and resuspended in PBS to prepare a 2% (v/v) RBC suspension. PEC NPs (12.5-400 μg/mL) were incubated with the RBC suspension at 37 °C for 4 h under gentle agitation. Positive and negative controls were established using 1% Triton X-100 (100% hemolysis) and PBS (0% hemolysis), respectively. Following incubation, the mixtures were centrifuged at 1,500 × g for 10 min. The absorbance of the supernatants was measured at 540 nm using a microplate reader (Synergy H1, BioTek Instruments, Inc.). The hemolysis rate (%) was calculated as:

$$\text{hemolysis rate (\%)} = \frac{OD_{Sample} - OD_{Negative\ control}}{OD_{Positive\ control} - OD_{Negative\ control}} \times 100\%$$

Where OD_{Sample}, $OD_{Positive\ control}$, $OD_{Negative\ control}$ and denote the absorbance values of the test sample, positive control, and negative control, respectively. All experiments were performed in triplicate.

***In Vivo* and *Ex Vivo* Imaging Procedure**

Tumor-bearing BALB/c mice implanted with CT26 tumors were randomly allocated into two experimental groups (n = 3 per group). Both formulations were administered via tail vein injection at a dosage of 20 mg·kg^{-1} body weight. Subsequently, whole-body fluorescence imaging was performed at predetermined time points using a Spectrum imaging system (Tanon ABL-X6, China) with mice under 2.5% isoflurane anesthesia. Following the final imaging session, animals were deeply anesthetized and subjected to transcardial perfusion with ice-cold phosphate-buffered saline (PBS, pH 7.4). Major organs (heart, liver, spleen, lungs, kidneys, muscle) and tumors were surgically excised, rinsed with PBS, and placed on a black imaging tray for *ex vivo* fluorescence imaging under identical instrument settings to the *in vivo* procedure.

Establishment of CT26 tumor model and therapeutic effect

CT-26 tumor-bearing mice were randomly divided into 7 groups. When the tumor volume reached 50 - 100 mm^3, the mice were administered PBS, aPD-1, PDPA, EC, PES, PEC and PEC + aPD-1 (n = 5, anti-PD-1 100 μg/mouse, PDPA 2 mg/mouse, EC 60 μg/mouse, PEC nanoparticles were administered at an EC-matched dose of 60 μg/ mouse). Different nanoparticles were intravenously injected every 3 days (on day 0, 4, 7 and 10, total 4 doses, injection volume: 100 μL), and PD-1 antibody was intraperitoneally injected every 3 days (on days 1, 5 and 8, total 3 doses, injection volume: 50 μL). Tumor volume and body weight were recorded every other day. At the end of the treatment, all mice were euthanized, and then lymph nodes, spleen and tumor tissues were collected for subsequent immune effect studies.

DC maturation and T cell activation in vivo

Mouse lymph nodes were collected and grinded into single-cell suspension, stained with anti-CD80, anti-CD86, anti-CD40 and anti-CD11c antibodies. Flow cytometry was performed to detect the expression of co-stimulatory molecules CD80, CD86 and CD40.

Macrophage polarization induced by nanoparticles

M1-type macrophages exert anti-tumor immunity, while M2-type macrophages promote tumor growth. To assess the proportion of tumor-infiltrating macrophages, we sorted macrophages from tumor tissue using anti-F4/80 antibodies, and CD86 and CD206 antibodies labeled M1- and M2-type macrophages, respectively.

Expression of tumor-infiltrating IFN-γ^+ T cells

Tumor-infiltrating IFN-γ^+ T cells were significant for antitumor efficacy and immune response. A mouse tumor-infiltrating tissue lymphocyte isolate kit was performed to isolate tumor-infiltrating lymphocytes. Anti-CD3, anti-CD4, anti-CD8, anti-granzyme

B and anti-IFN-γ antibodies were used to measure the expression of tumor-infiltrating IFN-γ^+ T cells.

Pathological analysis by H&E staining

After all mice were sacrificed, tumors, hearts, livers, spleens, lungs and kidneys were collected and stained with hematoxylin-eosin (H&E), followed by microscopic observation.

Evaluation on ICD effects

To demonstrate the CRT eversion, CT26 cells were seeded in 6-well culture plates with slides at a density of 10^5 cells/well and incubated overnight. The cells were treated with PBS, $CuCl_2$, EC or PEC for 6 h and irradiated. After incubation, the cells were fixed and blocked with immunostaining blocking buffer for 2 h, and incubated with CRT antibody and subsequent fluorescent secondary antibody at 37 °C for 1 h, respectively. After washing with PBS and staining with DAPI, the slides were finally observed with fluorescence microscope.

ATP and HMGB-1 detection

CT26 cells were seeded in 6-well plates at a density of 2×10^5 cells/well and incubated overnight. Subsequently, they were treated with PBS, $CuCl_2$, EC or PEC for 6 h. The cells were then washed with PBS three times. The intracellular ATP content was measured by ATP assay kit and HMGB-1 assay kit according to the manufacturer's instructions.

Statistical Analysis

All the data are presented as Mean ± SD or SEM (n = 3 for in vitro experiments and n = 5 for in vivo experiments). Comparisons between two groups were analyzed using two sample student's t-test. Comparisons among several groups were analyzed using one-way analysis of variance. Survival curves were analyzed by Kaplan-Meier survival analysis using the log rank (Mantel-Cox) test. $p < 0.05$ was considered statistically

significant. Statistical significance was defined as *: $p < 0.05$, **: $p < 0.01$, ***: $p < 0.001$, ****: $p < 0.0001$ and ns indicated no significant differences ($p > 0.05$).

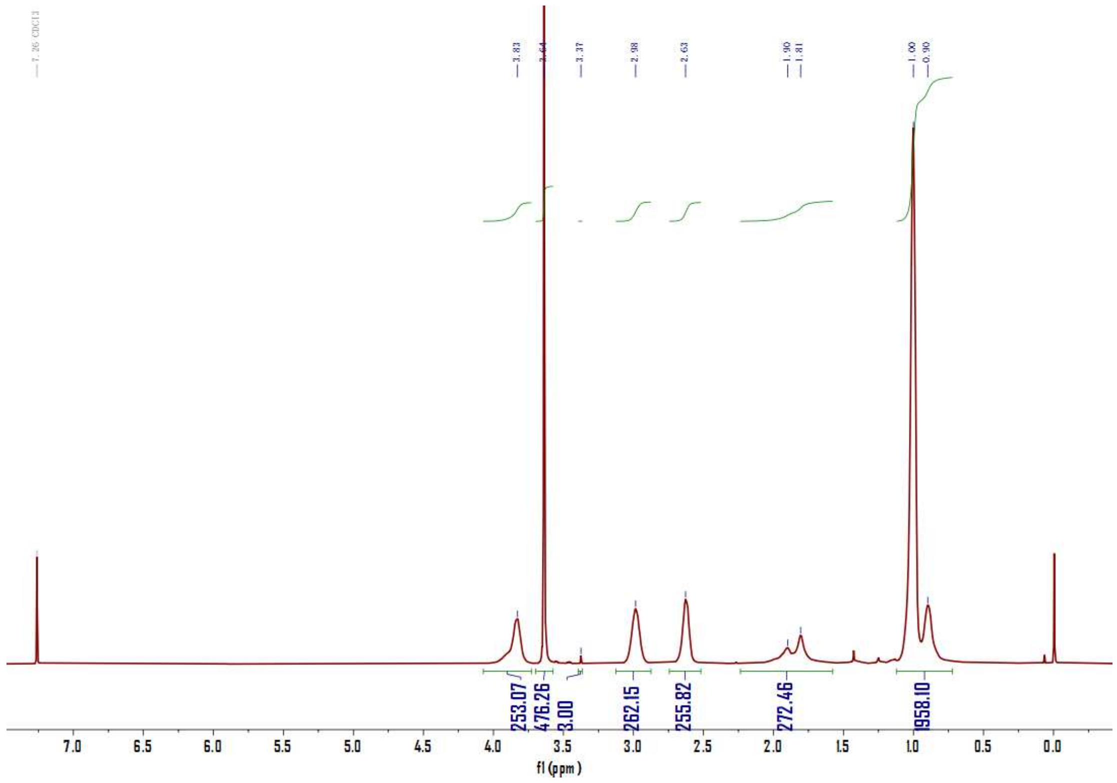

Figure S1. ¹H NMR spectra of PDPA in $CDCl_3$. ¹H NMR (400 MHz, $CDCl_3$) δ 3.83 (s, 253H), 3.64 (s, 476H), 3.38 (s, 3H), 2.99 (s, 262H), 2.63 (s, 256H), 2.24–1.58 (m, 272H), 1.01 (s, 1958H).

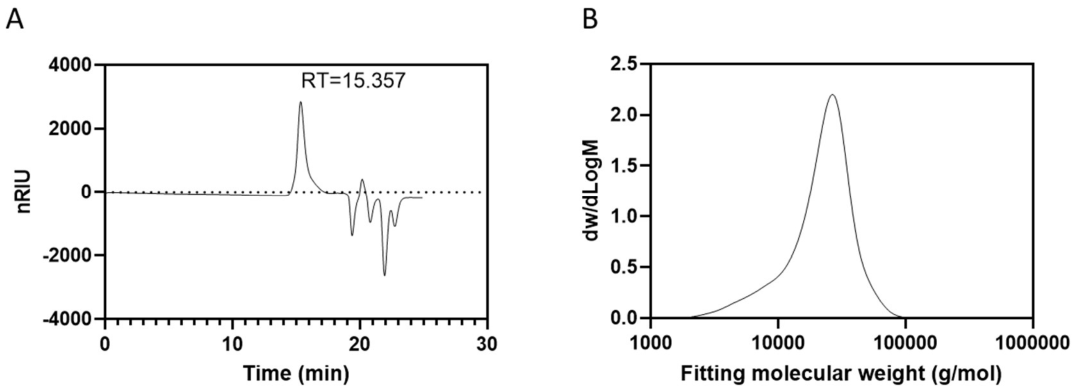

Figure S2. Chromatogram(A) and molecular weight(B) distribution of PDPA.

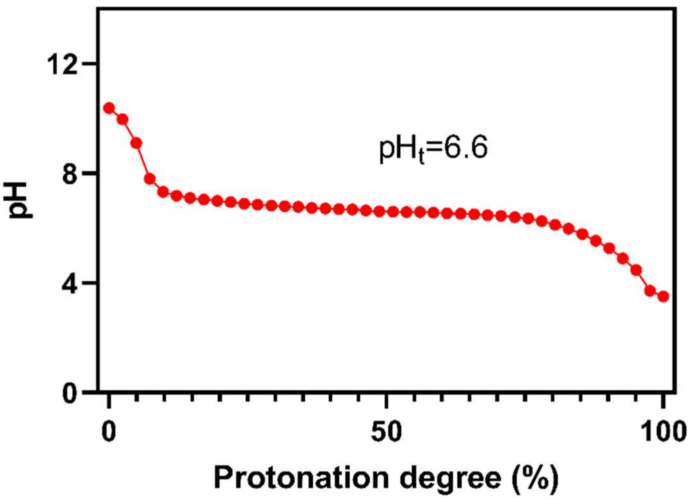

Figure S3. pH-titration curves of PDPA polymer.

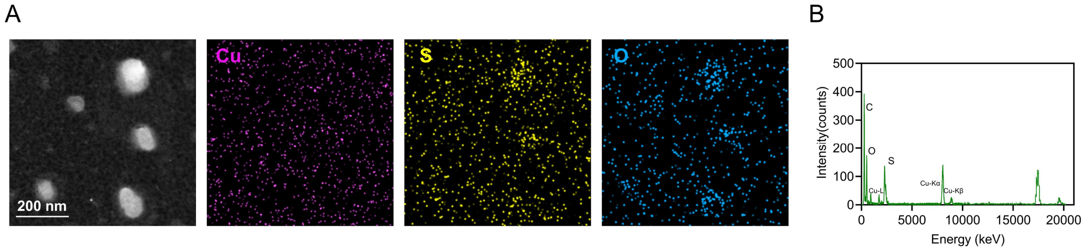

Figure S4. Representative elemental mapping image(A) and TEM-EDS(B) of PEC NPs.

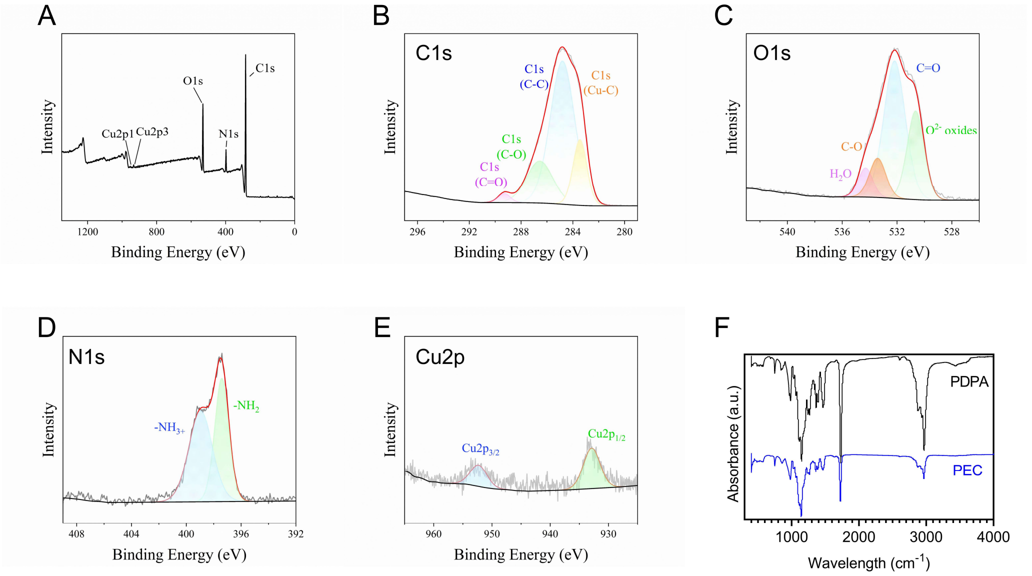

Figure S5. (A)XPS analysis of PEC NPs. High-resolution C 1s(B), O 1s(C), N 1s(D) and Cu2p(E) XPS spectra of PEC NPs. (F) FTIR spectra of PEC NPs.

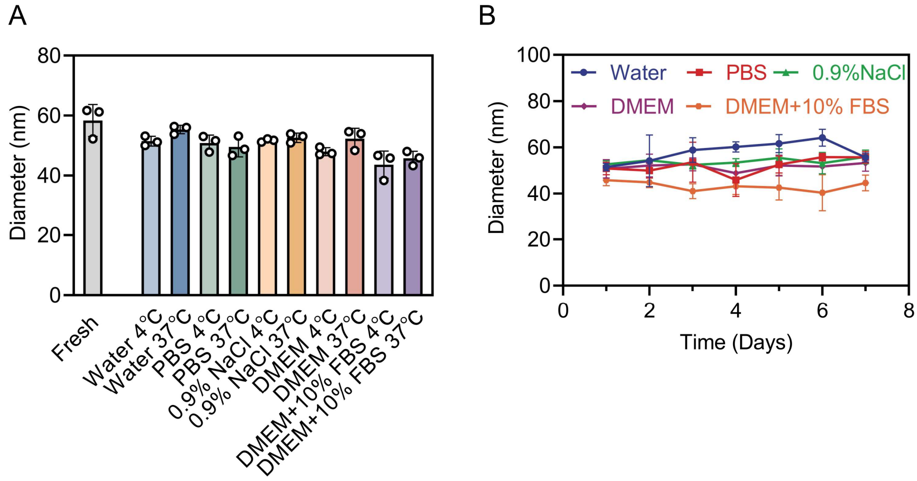

Figure S6. Temperature- and time-dependent stability of PEC NPs in diverse biological media (Media tested: ultrapure water, PBS (pH 7.4), 0.9% NaCl, DMEM, and DMEM supplemented with 10% FBS).

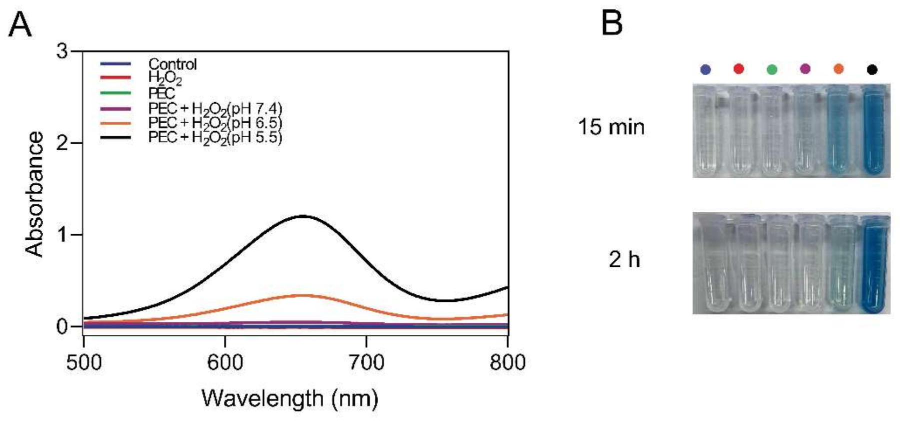

Figure S7. (A) pH-dependent oxidation of TMB by PEC NPs at 15 min. (B) Photos of samples from different groups at 15 min and 2 h.

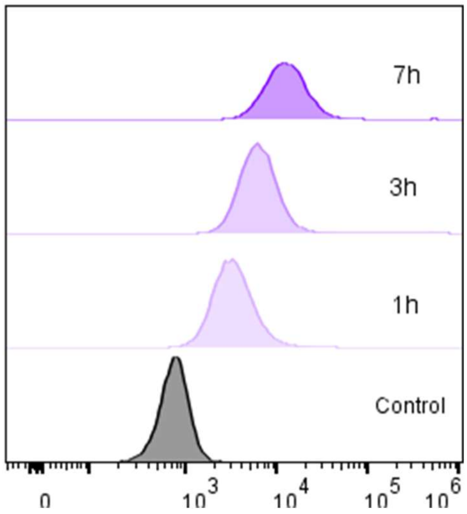

Figure S8. The cellular uptake of PEC^{Cy5} NPs at different time.

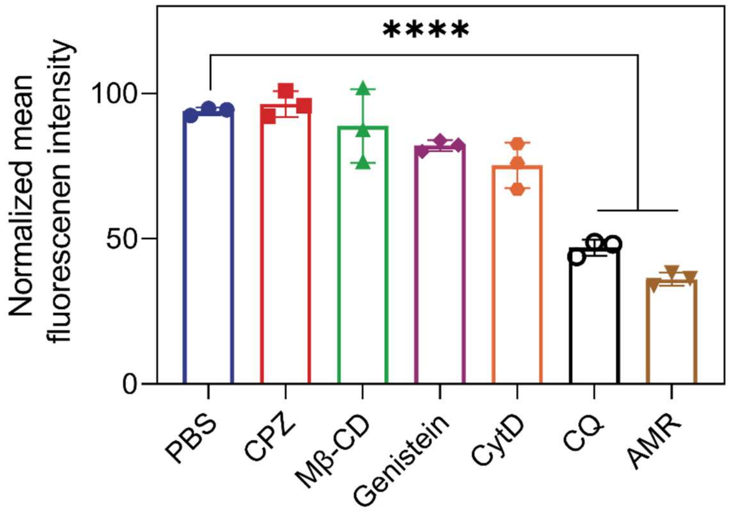

Figure S9. Relative cellular uptake level of cells treated with specific pathway inhibitors and PEC^{Cy5} NPs.

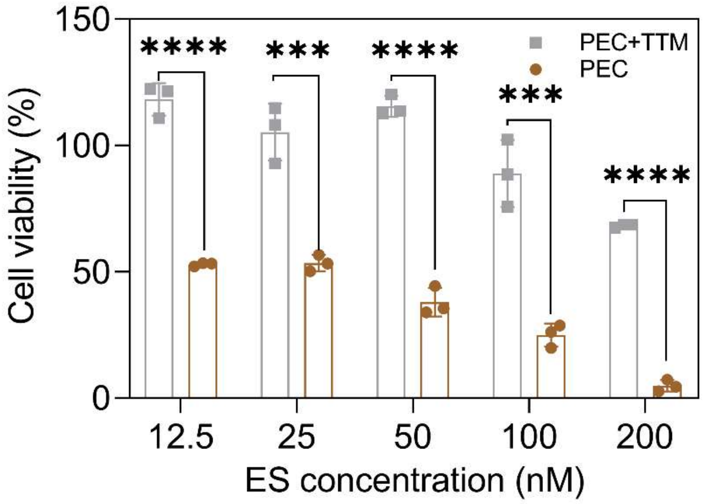

Figure S10. Cytotoxicity assessment of PEC NPs on CT26 cells with or without TTM.

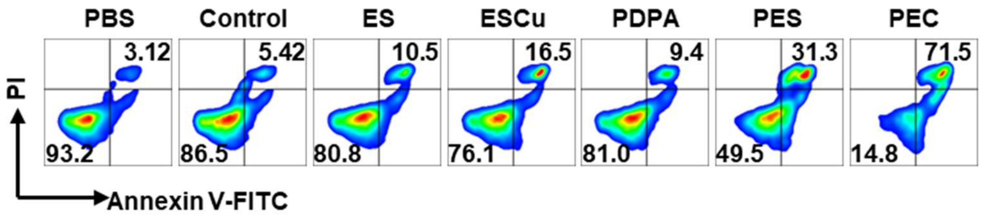

Figure S11. Flow cytometry analysis of the apoptotic percentage of CT26 under various treatments.

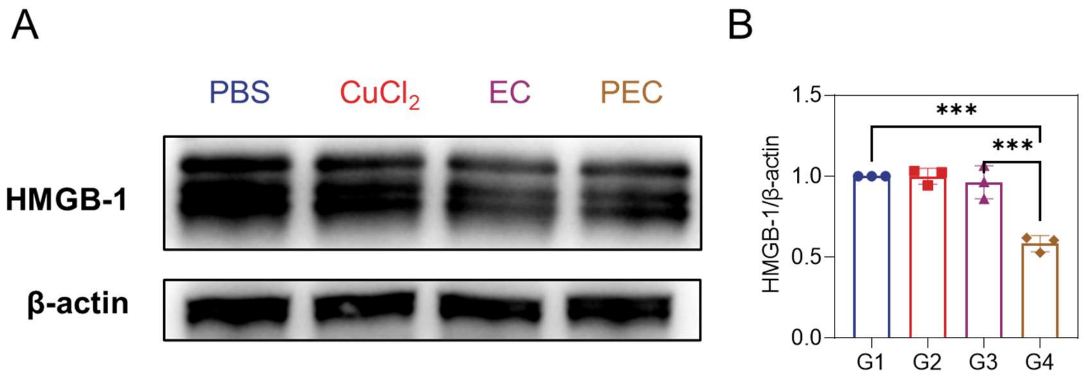

Figure S12. Western blot analysis of HMGB-1 expression in CT26 cells after different treatments.

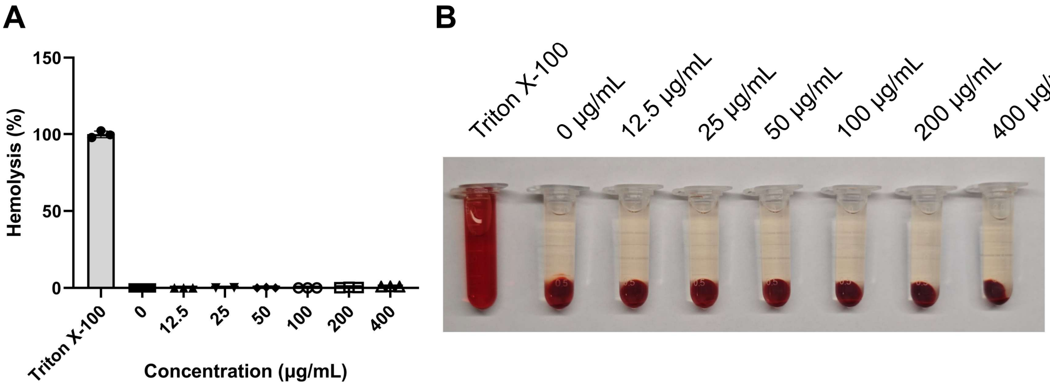

Figure S13. Hemolysis rate(A) and image(B) of PEC series concentration.

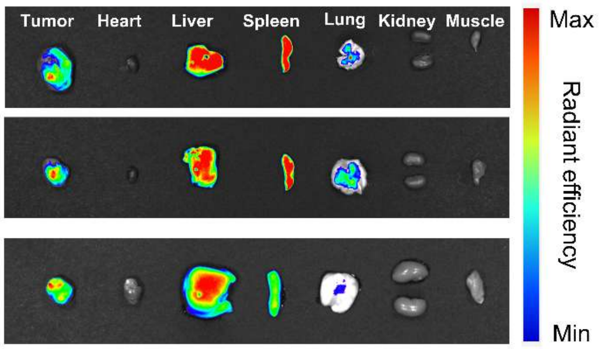

Figure S14. *Ex vivo* imaging of major organs of tumor-bearing mice at 24 h post-injection of PEC NPs^{Cy5}.

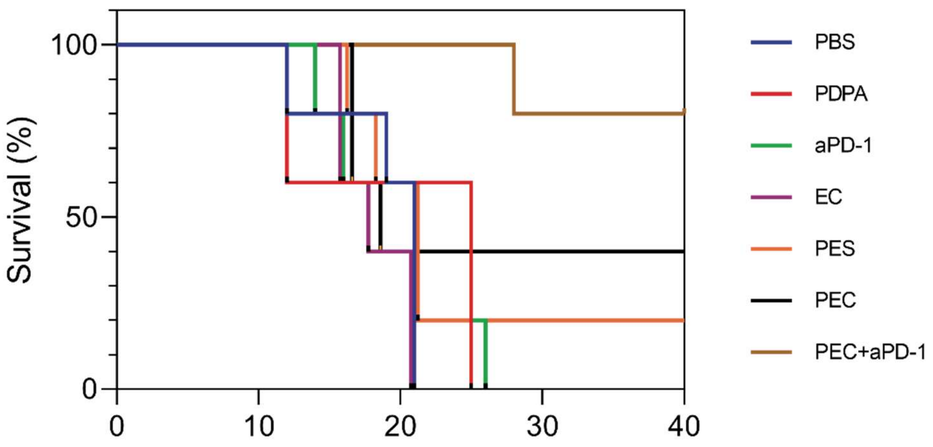

Figure S15. Survival time of CT26 tumor-bearing BALB/c mice with various treatments.

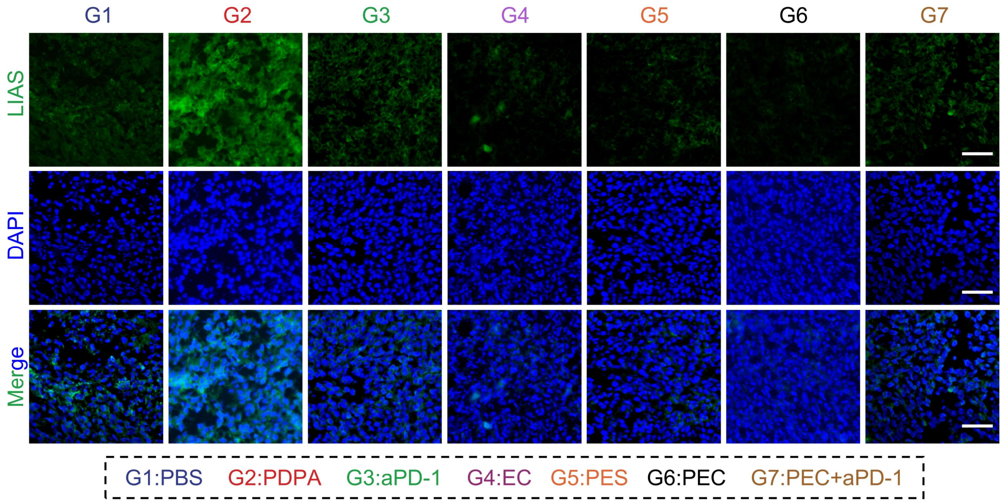

Figure S16. Validation of cuproptosis by LIAS staining in tumor after various treatments. A significantly decreased expression level of LIAS was detected in the tumor tissues treated with PEC NPs, PES NPs, and PEC NPs + aPD-1. Scale bar: 50 μm.

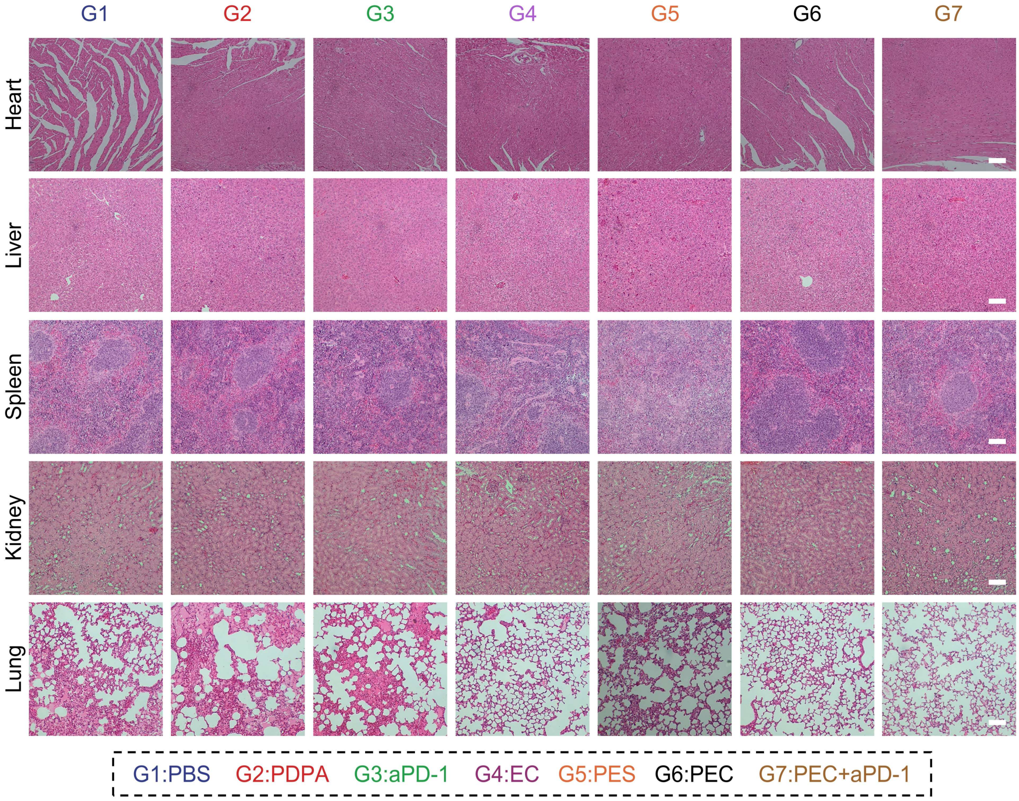

Figure S17. H&E staining of the main organ tissues (heart, liver, spleen, lung and kidneys) in different treatments. Scale bar: 250 μm.

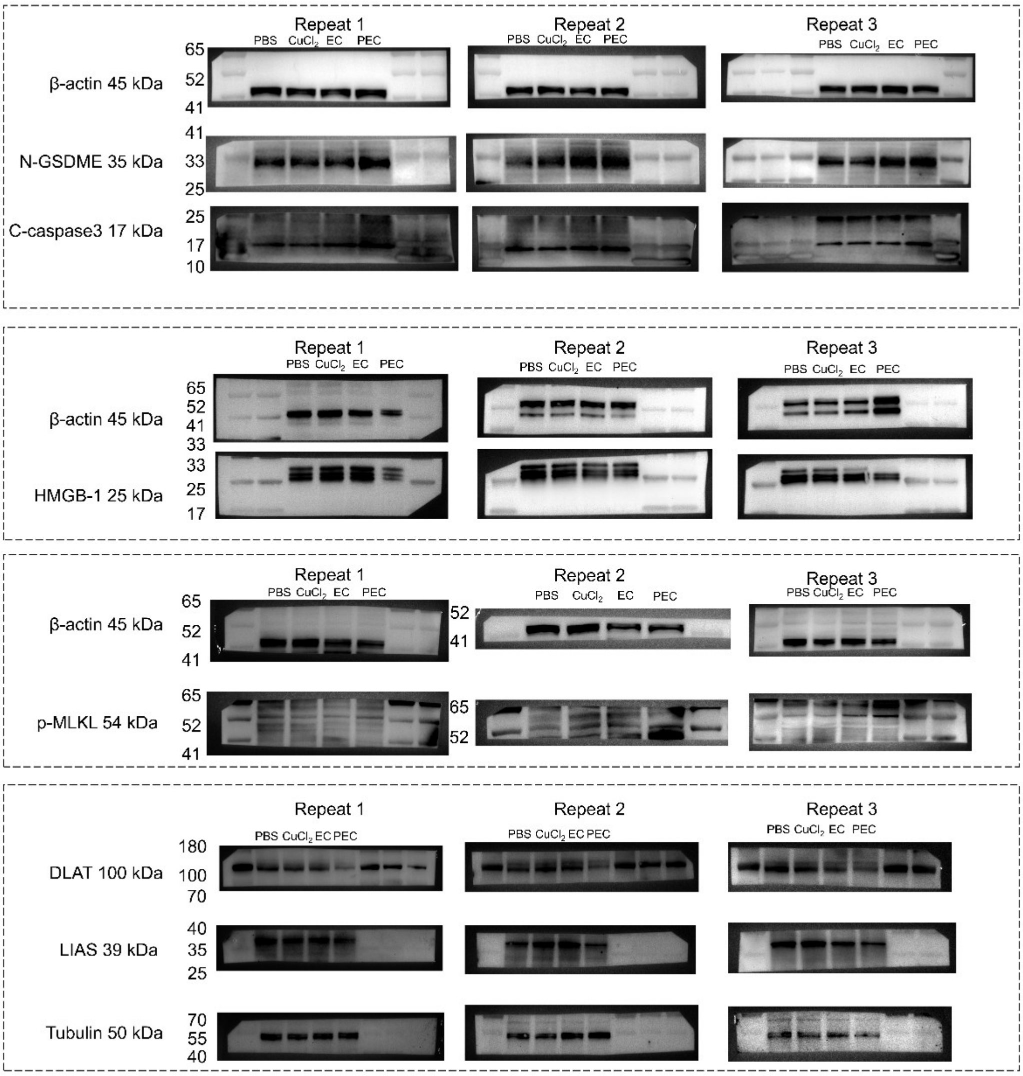

Figure S18. Original western blot for three repeats.

Table S1. Molecular weight information of $PDPA_{120}$

RT(min)	M_p(g/mol)	M_n(g/mol)	M_w(g/mol)	M_z(g/mol)	M_{z+1}(g/mol)	PDI
15.357	26519	17220	24753	31159	37438	1.437456
